# Discrimination by parity is a prerequisite for assessing induction of labour outcome – cross-sectional study

**DOI:** 10.1186/s12884-020-03334-8

**Published:** 2020-11-23

**Authors:** Branko Denona, Michael Foley, Rhona Mahony, Michael Robson

**Affiliations:** 1 National Maternity Hospital, Dublin, Ireland; 2 St. Luke’s General Hospital, Kilkenny, Ireland

**Keywords:** Nulliparous women, Multiparous women, Induction of labour

## Abstract

**Background:**

To demonstrate that studies on induction of labour should be analyzed by parity as there is a significant difference in the labour outcome among induced nulliparous and multiparous women.

**Methods:**

Obstetric outcome, specifically caesarean section rates, among induced term nulliparous and multiparous women without a previous caesarean section were analyzed in this cross-sectional study using the Robson 10 group classification for the year 2016.

**Results:**

In the total number of 8851 women delivered in 2016, the caesarean section rates among nulliparous women in spontaneous and induced labour, Robson groups 1 and 2A, were 7.84% (151/1925) and 32.63% (437/1339) respectively and among multiparous (excluding those women with a previous caesarean section), Robson group 3 and 4A were 1%(24/2389) and 4.37% (44/1005), respectively. Pre labour caesarean rates for nulliparous and multiparous women, Robson groups 2B and 4B (Robson M, Fetal Matern Med Rev, 12; 23–39, 2001) were 3.91% (133/3397) and 2.86% (100/3494), of the respective single cephalic cohort at term.

**Conclusion:**

The data suggests that studies on induction of labour should be analyzed by parity as there is a significant difference between nulliparous and multiparous women.

## Background

The overall induction of labour rate in Ireland is 25% [1]. The induction rate among single cephalic nulliparous women, ≥ 37 weeks of gestation cohort group has increased in our hospital from 17.5% when the Robson classification [2] was introduced in 1994 to 39.41% in 2016 (Table 1). This increase in the induction rate is due to a variety of reasons including, ‘prolonged pregnancy’, gestational diabetes, cholestasis in pregnancy, patient’s request; indications which are usually recurrent and will, most likely, present a problem in subsequent pregnancies for women who have been delivered by caesarean section for failed induction in their first pregnancy.

**Table 1:** Robson 10 group classification and the results for NMH 2016

Group	Description	Total numbers of caesarean sections (2303/8851)	Contribution of the group in overall hospital population	Caesarean section rate within each group	Contribution of each group to overall CS (26%)
1	Nulliparous, single cephalic, > = 37 weeks, in spontaneous labour	151/1925	21.74%	7.84%	1.70%
2	Nulliparous, single cephalic, > = 37 weeks, induced and CS before labour	570/1472	16.63%	38.72%	6.43%
2A	Nulliparous, single cephalic, > = 37 weeks, induced	437/1339	15.12%	32.63%	4.93%
2B	Nulliparous, single cephalic, > = 37 weeks, CS before labour	133	1.50%	100%	1.50%
3	Multiparous (excluding prev. CS), single cephalic, > = 37 weeks, in spontaneous labour	24/2389	26.99%	1%	0.27%
4	Multiparous (excluding prev. CS), single cephalic, > = 37 weeks, induced and CS before labour	144/1105	12.48%	13.03%	1.62%
4A	Multiparous (excluding prev. CS), single cephalic, > = 37 weeks, induced	44/1005	11.35%	4.37%	0.49%
4B	Multiparous (excluding prev. CS), single cephalic, > = 37 weeks, CS before labour	100	1.12%	100%	1.12%
5	Previous CS, single cephalic, > = 37 weeks	821/1069	12.07%	76.80%	9.27%
6	All nulliparous breeches	162/171	1.93%	94.73%	1.83%
7	All multiparous breeches (including prev. CS)	115/124	1.40%	92.74%	1.29%
8	All multiple pregnancies (including prev. CS)	119/187	2.11%	63.63%	1.34%
9	All abnormal lies (including prev. CS)	30	0.33%	100%	0.33%
10	All single cephalic,<=36 weeks (including prev. CS)	167/379	4.28%	44.06%	1.88%

A PubMed search (years 2010–2016) for publications on induction of labour was performed to determine how many abstracts mentioned parity. A search produced 404 abstracts of which only 77(19.05%) specifically stated that the study was confined to nulliparous or multiparous women. Of the remaining, 136(33.66%) mentioned parity as a variable in the analysis of results and 191 (47.27%) did not mention parity at all.

From clinical perspective, induction of labour in nulliparous women carries higher risk of caesarean section due to failed process of induction compared to multiparous women and we investigated our data collected over a period of 1 year to see our results.

## Methods

This was a cross-sectional study of data collected at the time of delivery on a computer database at the National Maternity Hospital, Dublin in year 2016. Caesarean section rates for term single cephalic nulliparous and multiparous women without previous uterine scar, were taken from data published in the annual hospital report. The indication for induction were classified under 6 headings: preeclampsia (hypertension and proteinuria)/ hypertension, postdates > = 42 weeks, SROM, maternal reasons/pains, fetal reasons (IUGR, reduced liquor, GDM, obstetric cholestasis and others.) and nonmedical reasons (maternal request for postdates in prolonged pregnancy but not > = 42 weeks) (Table 4).

The classification of caesarean section, performed during induction process or after labour was diagnosed, was classified as fetal reasons (without the use of oxytocin) and dystocia (which was further sub classified) [3, 4] (Table 5).

Following admission for induction of labour a CTG was performed and the cervix was assessed by an experienced obstetrician. When the cervix was thought to be favorable artificial rupture of the membranes was performed (ARM) and an oxytocin infusion was commenced the following day if labour had not commenced.

When the cervix was deemed to be unfavorable, a prostaglandin PGE2 intravaginal gel was administrated and repeated if necessary, in 6 h provided the repeat CTG were normal. Number of women were treated with Propess instead of PGE2 gel by the same principle. If labour had not commenced by the following day, the induction process was repeated, ARM or prostaglandin gel. When there was no change in cervical status after 2 days of induction process, a caesarean section was performed but was included in Robson group 2A or 4A. Bishop score is not regularly used in our hospital, so the changes in cervix are noted in length and dilatation measured in centimeters and progress evaluated on this observations.

## Results

The induction rate among single cephalic nulliparous women at term (≥ 37 weeks of gestation) increased from 17.5% in 1994 to 39.41% in 2016 and caesarean section rate increased from 21.5% (97/451) to 32.63% (437/1339) respectively. The induction rate among term multiparous women without previous caesarean section (group 4A) increased from 17.02% in 1994 (626/3677) to 28.76% (1005/3494) in 2016 and the respective caesarean section rates were 5.11% (32/626) in 1994 and 4.37% in 2016 (44/1005).

There were 8851 women delivered in 2016 of whom 3397 were in Robson group 1 and 2. Among these 56.66% (1925/3397) went into spontaneous labour, 39.41% (1339/3397) were induced and 3.91% (133/3397) had pre labour caesarean section. The corresponding number for multiparous women in Robson group 3 and 4 were 68.37% (2389/3494), 28.76% (1005/3494) and 2.86% (100/3494). Excluded from analysis from multiparous women group were 1069 women with at least 1 previous caesarean section and a single cephalic pregnancy (Robson group 5).

Patient demographics, method of induction, obstetric and neonatal outcome are shown in Table 2. Of note, group 4A were significantly older and had significantly larger babies. However, the percentage of obese women (BMI = > 30) was similar. More nulliparous women required prostaglandins combined with oxytocin infusion for induction (Table 2).

**Table 2:** Patient demographics, method of induction, maternal and fetal outcome (NMH 2016)

	Group 2A	Group 4A	95% CI	*p*-value
Age > =35 years	439/1339 (32.78%)	531/1005 (52.83%)	15.9 to 23.9	*P* < 0.0001
BMI= > 30	166/1339 (12.39%)	145/1005 (14.42%)	−0.7 to 4.8	*P* = 0.1577
Prostaglandin gel/Propess	666/1339 (49.73%)	348/1005 (34.62%)	11 to 19	*P* < 0.0001
Oxytocin	961/1339 (71.76%)	323/1005 (32.13%)	35.8 to 43.3	*P* < 0.0001
Artificial rupture of membranes	882/1339 (65.87%)	851/1005 (84.67%)	15.3 to 22.1	*P* < 0.0001
Fetal blood sampling	380/1339 (28.37%)	68/1005 (6.76%)	18.6 to 24.4	*P* < 0.0001
Vaginal operative delivery	392/1339 (29.27%)	57/1005 (5.67%)	20.7 to 26.3	*P* < 0.0001
Full dilatation caesarean section	34/1339 (2.53%)	2/1005 (0.19%)	1.4 to 3.2	*P* < 0.0001
PPH= > 1000 ml	63/1339 (4.70%)	27/1005 (2.68%)	0.4 to 3.5	*P* = 0.0127
HIE	5/1339 (0.37%)	0/1005 (0%)	−0.1to 0.9	*P* = 0.0448
Blood transfusion	45/1339 (3.36%)	9/1005 (0.89%)	1.3 to 3.6	*P* = 0.0001
OASIS	29/1339 (2.16%)	12/1005 (1.19%)	−0.1 to 2.1	*P* = 0.0694
Apgar< 7 at 5 min.	20/1339 (1.49%)	8/1005 (0.79%)	−0.2 to 1.5	*P* = 0.1235
Cord pH < 7.0	5/1339 (0.37%)	2/1005 (0.19%)	−0.3 to 0.7	*P* = 0.3920
Admission to Neonatal unit	405/1339 (30.24%)	145/1005 (14.42%)	12.4 to 19	*P* < 0.0001
Babies> = 4 kg	242/1339 (18.07%)	264/1005 (26.26%)	4.8 to 11.6	*P* < 0.0001
Episiotomy	570/1339 (42.56%)	85/1005 (8.45%)	30.8 to 37.1	*P* < 0.0001
Epidural	1023/1339 (76.40%)	517/1005 (51.44%)	21.1 to 28.7	*P* < 0.0001
Electronic monitoring	1238/1339 (92.45%)	913/1005 (90.84%)	−0.5 to 4	*P* = 0.1384
Length of labour > 12 h	103/1339 (7.69%)	9/1005 (0.89%)	5.2 to 8.4	*P* < 0.0001

The caesarean rates among nulliparous women in spontaneous and induced labour, groups 1 and 2A, were 7.84% (151/1925) and 32.63% (437/1339) and among multiparous, group 3 and 4A were 1% (24/2389) and 4.37% (44/1005), respectively (Table 3).

**Table 3:** Caesarean section rates among nulliparous and multiparous women in spontaneous and induced labour (NMH 2016)

	Spontaneous labor	Induced labour
Nulliparous women	7.84% (151/1925)	32.63% (437/1339)
Multiparous women	1% (24/2389)	4.37% (44/1005)

Overall, the caesarean section rate by indication was lowest in both groups when the indication for induction was for fetal reasons or maternal pains. Among nulliparous women, the highest caesarean section rate by indication were for postdates pregnancies (= > 42 weeks) and for nonmedical reasons and late pregnancies < 42 weeks (Table 4).

**Table 4:** Indications for induction among group 2A and 4A and the associated caesarean section rates (NMH 2016)

	Group 2A (*n* = 1339)	CS rate for group 2A	Group 4A (*n* = 1005)	CS rate for group 4A
Fetal reasons	32.48% (435)	26.43% (115/435)	27.56% (277)	4.33% (12/277)
SROM	24.42% (327)	33.02% (108/327)	14.72% (148)	6.08% (9/148)
Postdates (> = 42 weeks)	14.86% (199)	44.22% (88/199)	12.83% (129)	3.87% (5/129)
PET/hypertension	11.87% (159)	29.55% (47/159)	5.87% (59)	6.77% (4/59)
Maternal reasons (including pains)	9.03% (121)	26.44% (32/121)	19.20% (193)	3.10% (6/193)
Nonmedical reasons/dates (< 42 weeks)	7.31% (98)	48.0% (47/98)	19.80% (199)	4.02% (8/199)
Total	39.4%(1339)	32.63% (437/1339)	28.8%(1005)	4.37% (44/1005)

The indications for caesarean sections are shown in Table 5 and as expected, the main difference between group 2A and 4A was the number indicated for dystocia and suspected fetal distress (Table 5).

**Table 5:** Indication for cesarean delivery among Robson group 2A and 4A

	Group 2A (*n* = 1339)	Group 4A (*n* = 1005)
Fetal reasons	7.5%(100)	1.0%(10)
Dystocia/IUA/ITT/FI	9%(121)	0.8%(8)
Dystocia/IUA/ITT/OC	5%(68)	0.7%(7)
Dystocia/IUA/PR	8.1%(108)	1.4%(14)
Dystocia (no oxytocin)	1.1%(15)	0.1% (1)
Dystocia/EUA/CPD/POP	1.9%(25)	0.4% (4)
Total	32.6%(437)	4.4%(44)

## Discussion

As the number of inductions are seemingly increasing there is a realization that the most significant groups to study are groups 2A and 4A from the Robson classification; in particular, group 2A.

From our data, nulliparous women are three to four times more likely to be delivered by caesarean section when labour is induced. Despite every effort over the years to address this important clinical problem including ARM, oxytocin infusion and prostaglandin in a variety of combinations, it seems that induction of labour in nulliparous women remains a challenge. In delivery units that report lower caesarean section rates in nulliparous women who are being induced it is often associated with a much longer labour process something which is certainly not viewed positively by all women and may have higher postpartum hemorrhage rates. In addition, it is not easy to audit the results in that induction of labour needs to be compared with expectant management and not directly with spontaneous labour [4].

On the other hand, multiparous women who have previously delivered vaginally and without a cesarean section are the lowest risk of our obstetric population with a low cesarean rate birth in spontaneous and induced labour, looking at our results. Achieving vaginal delivery in nulliparous women therefore seems important. The cesarean rate for nulliparous by indication for induction is important and the rate seems highest in those induced in late pregnancy either for the strict definition of > = 42 weeks or those induced for nonmedical reason or dates < 42 weeks.

We fully appreciate limitations of this study which include possible wrong allocation of certain number of patients in observed groups which would be non-significant for the overall result, difference between examiners of patients and slight adjustments in induction of labour process.

## Conclusions

As we search for new methods for induction of labour we believe that the data presented here provides evidence that trials on labor induction should be more focused on nulliparous women. We have not attempted to address the separate and contentious problem of labour induction in women with a previous cesarean section; avoiding the first cesarean section seems to be the only solution, either by trying to avoid induction or prelabour caesarean section when possible; or by introducing new techniques to increase the success of induction of labour.

## Data Availability

All the data used in this study is published in hospital annual report and is available from the hospital on reasonable request.

## Abbreviations

AROM: Artificial rupture of membranes
SROM: Spontaneous rupture of membranes
IUGR: Intrauterine growth restriction
GDM: Gestational diabetes mellitus
IUA: Inefficient uterine activity
EUA: Efficient uterine activity
ITT: Inability to treat
FI: Fetal intolerance
OC: Over contracting
PR: Poor response
CPD: Cephalopelvic disproportion
POP: Persistent occiput posterior
